# Prospective policy analysis: how an epistemic community informed policymaking on intentional self poisoning in Sri Lanka

**DOI:** 10.1186/1478-4505-8-19

**Published:** 2010-06-17

**Authors:** Melissa Pearson, Zwi B Anthony, Nicholas A Buckley

**Affiliations:** 1 South Asia Clinical Toxicology Research Collaboration, Faculty of Medicine, University of Peradeniya, Sri Lanka; 2 School of Public Health and Community Medicine, Faculty of Medicine, The University of New South Wales, Sydney Australia; 3 Prince of Wales Hospital Clinical School, The University of New South Wales, NSW Australia

## Abstract

**Background:**

Policy analysis is often retrospective and not well suited to helping policy makers decide what to do; in contrast prospective policy analysis seeks to assist in formulating responses to challenging public policy questions. Suicide in Sri Lanka is a major public health problem, with ingestion of pesticides being the primary method. Previous policy interventions have been associated with reduced mortality through restricting access to the most toxic pesticides. Additional means of reducing access are still needed.

**Methods:**

The prospective policy analysis comprised two stages. The first used a consensus activity within a well defined policy community to generate and frame policy options. The second broadened the analysis to include other stakeholders. We report the consensus activity with seven actors from agriculture, health, and academia. Policy options were identified through two rounds of discussion along with ratings by each participant on their degree of support for each option. Data were analysed quantitatively and discussions analysed with Nvivo 8 to code prominent and recurrent themes.

**Results:**

The main finding was the strong support and consensus for two proposals: further regulation of pesticides and the novel idea of repackaging pesticides into non-lethal doses. Participants identified several factors that were supportive of future policy change including a strong legislative framework, good links between agriculture, health and academia, and a collaborative relationship with industry. Identified barriers and potential threats to policy change included political interference, difficulties of intersectoral collaboration, acceptability of options to the community, difficulty of implementation in rural communities and the challenge of reducing mortality.

**Conclusions:**

The development and consideration of policy options within this epistemic community reflected an appreciation and understanding of many of the factors that can facilitate or thwart policy change. The understanding of context, evidence and ideas, implementation and impact influenced how the participants considered and rated the options. Use of epistemic community actors identified the level of support for each option, helped elaborate the particularities of context, as well as the power and influence of ideas. Further examination of the potential barriers and opportunities for these options will determine if broader consensus, involving a wider range of stakeholders, can be achieved and policy change promoted.

## Background

### Suicide in Sri Lanka

Suicide in Sri Lanka is a major public health problem. In 1995 the country had the highest rate of suicide worldwide (approximately 47 per 100,000) [1]. Recent analysis of the incidence of suicide has shown a substantial decline from the peak of 47 in 1995 (male 80 and female 28) to 24 per 100,000 in 2005 (male 37 and female 10) [2]. Young people are over-represented in admission rates following self-harm. In 2000, 60% of all self-harm admissions were aged 16-25 [3]. Of all the suicide deaths recorded, between 60 - 80% are caused by the ingestion of pesticides [4]. In many rural districts of Sri Lanka the most common cause of death from any cause is from the intentional ingestion of pesticides [5]. While deaths from suicide overall have been falling, there remains a considerable burden of intentional self poisoning [6-9].

Policy responses to the problem of suicide in Sri Lanka included establishing a Presidential Committee, legislative changes, and improved clinical management. The Presidential Committee (formed in 1996) developed a National Suicide Prevention Strategy in 1998 with the goals of:

• Reducing easy access to lethal methods.

• Promoting research on reducing the lethality of pesticides in use.

• Educating the public on less harmful use of pesticides

• Creating a culture which discourages suicides

• Ensuring survival after poisoning

• Removing legal barriers to the correct handling of those at risk [7].

Since the inception of the Control of Pesticides Act 1980 the Department of Agriculture has embarked on a concerted programme to regulate the most toxic pesticides. These restrictions have been associated with considerable success, as described above, in reducing the overall mortality from intentional self-poisoning [10,11]. In 2008 the Department of Agriculture announced a phased withdrawal of three pesticides (Paraquat, Dimethoate and Fenthion) based on strong evidence of the high death rates associated with their misuse in rural communities. Following these decisions there was concern from stakeholders about which interventions should, at that stage, be pursued and how best to continue to reduce mortality associated with the intentional ingestion of pesticides.

### Health Policy Analysis

Health policy analysis is a reasonably developed field in industrialized nations but only a relatively small body of work has emerged from low and middle income countries [12,13]. Lessons to date have recently been synthesized [14,15]. Since the early 1990's there has been a growing interest in why policies do not achieve their anticipated impact and much research has moved away from analysis of the content of policy to focus more on the political dimensions of policymaking and implementation [16,17]. Most reported health policy analyses have been retrospective case studies looking at the success or failure of past policies [15], yet policymakers need prospective tools to help analyse strategies before they are implemented.

Health policy analysis draws on two prominent discourses, based on different disciplinary influences. While not mutually exclusive their traditions differ slightly with public policy analysis rooted in the social and political economy frameworks of public policy while evidence-informed policymaking is rooted in the scientific and health care domains and draws from, but refines, the evidence-based medicine traditions.

**Figure 1:**
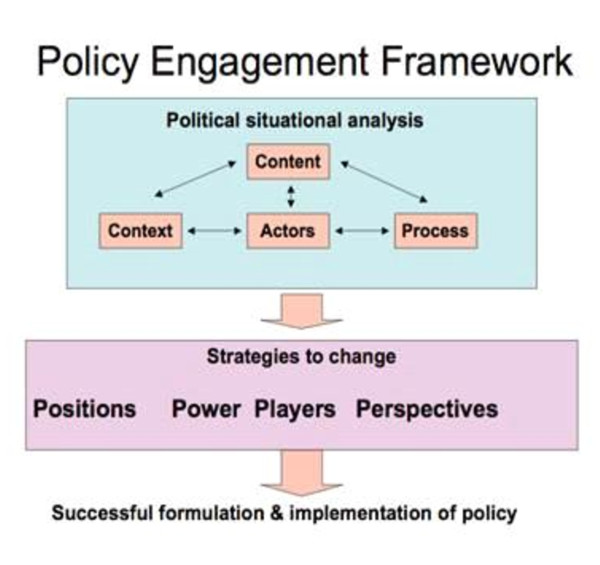
**Policy Engagement Framework**. Buse K, *et al:*2008 [39].

Within the health field a popular approach to examining policy is to utilise the 'policy triangle' - an elegant and simple tool which has been used extensively in low and middle income countries and identifies the content of policy, contextual factors, processes and actors as all deserving of attention [13]. Walt and Gilson suggest that these factors are intertwined and need to be systematically considered to achieve an holistic understanding [18]. Buse, an advocate of prospective policy analysis [19], has gone further to develop a policy engagement framework to prospectively analyse policy that incorporates strategies for change (see Figure 1). The policy engagement framework "offers a systematic approach to the ongoing collection, analysis and use of political information (e.g. concerning actors, their interests, institutions, ideas, and policy processes and context) that can alter the balance of power between those in support of and those resisting change by enabling pro-reformers to intervene more effectively in the policy process" [20]. This framework, like the political mapping approach of Reich [12], identifies the key domains of interest in analyzing policy prospectively.

**Figure 2:**
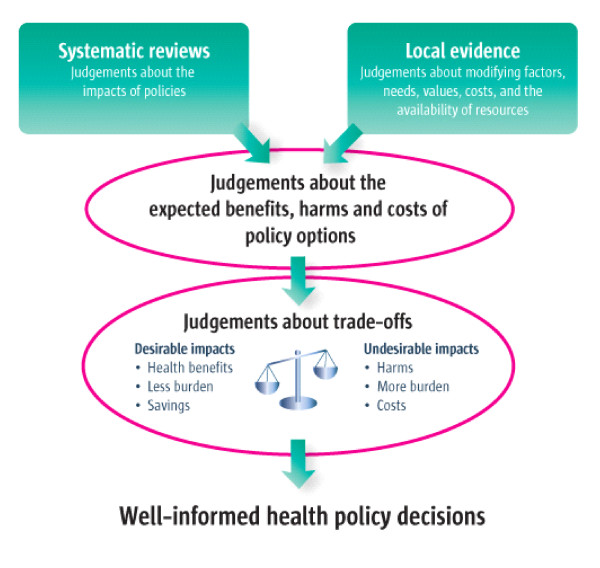
**Well informed policy decisions**. Oxman *et al*. 2009 [39].

In addition to these domains developed through the policy sciences traditions, there is increasing focus on the pathways to promote evidence-informed policy-making [21]. Recent publication of the SUPPORT tools for evidence-informed policymaking seeks to guide policymakers and the policy community to access better knowledge to support policy making. The 18 papers in the SUPPORT suite address four broad areas related to policymaking: supporting evidence-informed policymaking, identifying needs, finding and assessing information and implementation [22]. They propose that well informed policy decisions are made based on using systematic reviews and knowledge of local conditions in order to assess benefits, harms and costs and make judgements about trade-offs, as seen in Figure 2. While these tools are specifically geared towards policymakers' uses of evidence to inform policy, they recognise that evidence is only one factor in a multi-faceted process and acknowledge that this framework could be used to complement other strategies.

### Epistemic Communities

Lindblom argued that a good policy is one that commands consensus among policy makers and interest groups [23]. All the above frameworks and tools identify the importance of actors and stakeholders, reflecting the view of policy as the outcome of continuing interaction between diverse agendas, multiple stakeholders, and contested and ambiguous issues [24]. One important type of stakeholder network that is at times influential is an epistemic community - "a network of professionals with recognised expertise and competence in a particular domain and an authoritative claim to policy-relevant knowledge in that domain or issue area" [25]. This group is tightly integrated, limited in number, with high continuity and members having roughly equal status and power [26]. Thus, working with an epistemic community to generate a range of options for further analysis may itself yield important insights into policy, as well as into how the epistemic community operates, perceives the problem(s) and proposed solutions.

## Methods

We adopted two approaches to this problem; the first part a consensus exercise with the epistemic community to develop a range of policy options, and the second applying analytic tools to specific policy proposals with a wider range of stakeholders. This article details the findings from the first approach.

Consensus methods were developed by the RAND Corporation in 1969 to bring scientific rigour into collective processes for decision-making. The underlying aim of consensus methods such as a Delphi method "is to determine the extent to which experts agree about a certain issue" [27]. The consensus exercise employed here sought to generate policy options to reduce deaths from intentional poisoning with pesticides and used a modified Delphi technique that included four phases. The first and second phase involved the generation of a range of options from the literature and discussion between panellists, followed by a first round of voting. The third phase included feedback of the results from the first round of voting, a discussion of where consensus and dissensus were present and finally a second round of voting and ranking of options. A face-to-face Delphi technique was used as group processes have been found to be superior to non-group processes in terms of the number, quality and generation of unique ideas [28]. This technique was further supported by the presence, willingness and feasibility of organising known experts in the field to participate in a group process. The exercise aimed to develop a range of options to address the problem of the use of pesticides in suicide and to assess the feasibility and desirability of each to better control this hazard.

The exercise was facilitated by a non-voting established topic expert (NB) who was well known to the majority of participants, in keeping with good practice in consensus exercises. The panel members were selected from the existing policy community and included two Department of Agriculture officers, two international toxicologists with significant experience in Sri Lanka, one international public health academic with experience in Sri Lanka, one local academic actively involved in the field for the past 20 years and one local senior clinician/researcher. Evidence has shown that while panel size is not particularly important, attention to panel composition is crucial [29,30]. The generation of suggested panel members was guided by the local study review team to ensure an appropriate range of informants. All panel members knew each other and had collaborated on numerous previous projects, but their 'base' was in different disciplines and institutions.

Data were analysed both quantitatively and qualitatively. Analysis of the consensus level and the direction of the consensus was based on the method developed by de Loe [28] for data analysis of Delphi policy exercises shown in table 1. This system describes the level of consensus between categories as well as where the support is located with a high level of consensus defined as 70% of ratings in one agreement category or 80% in two contiguous categories. This was found to be superior to median and inter-quartile range analysis [28]. Qualitative data were analysed thematically using Nvivo 8 software. Data were coded for emergent and divergent themes. Ethical approval for the study was obtained from the University of New South Wales and University of Ruhuna.

**Table 1:** Definition and levels of consensus

Consensus level			Definition of consensus
**High**			70% of ratings in one agreement category or 80% in two contiguous categories*
**Moderate**			60% of ratings in one agreement category or 70% in two contiguous categories*
**Low**			50% of ratings in one agreement category or 60% in two contiguous categories*
**None**			Less than 50% of ratings in one agreement category or less than 60% of ratings in two contiguous categories*

* Contiguous agreement categories are 1 and 2 (in favour of consensus) or 3 and 4 (not in favour of consensus)[28]

## Results

### Generation of Options

Eight options were generated in the first round of discussion and described in Table 2. All of these options were supported from the literature however the generation of a novel suggestion that incorporated several ideas together, repackaging into non-lethal doses, was an innovative outcome from the discussion.

**Table 2:** Description of options generated through consensus activity

Restriction in access through bans	This utilises the current legislation (The Control of Pesticides Act 1980), working through the Pesticides Technical Advisory Committee (PeTAC). PeTAC is empowered to restrict the import of chemicals through a registration system. The Committee has restricted the import of all Class I chemicals and placed restrictions on 3 other chemicals since its inception.
**Reduce use of pesticides through promotion of IPM**	Integrated Pest/Vector Management are agricultural techniques developed to reduce the use of pesticides in farming. It is based on a model of participatory farming in which groups of farmers are taught to recognise pests and their natural enemies. It brought ecological principles and social scientific perspectives together to improve crop management techniques specifically in relation to pests (FAO 1994). Farmers are encouraged to wait for thresholds to be met and then to employ strategies to manage the pest. Pesticides are not proscribed but are discouraged. Farmers work together to identify the pests and to respond as a community. IPM has been widely promoted in Sri Lanka and Farmer Field Schools have been operating for the past 10 years. The Department of Agriculture supports the use of IPM and it is promoted through the National Agriculture Policy. Promotion of IPM/IPVM has been suggested as a means of reducing the amount of synthetic pesticides and therefore the availability in the community of toxic chemicals.

**Reduce use through promotion of bio-pesticides**	Bio-pesticides are pesticides that contain biological control organisms. It includes use of micro-organisms (such as bacteria, fungi or viruses) or products based on plant products (such as genetic engineering of plant to resist diseases) and biochemical pesticides that use natural substances (such as insect sex pheromones to ward off pests). There are high costs associated with the research and development of the modified seeds and input costs for farmers are high. The use of bio-pesticides has been limited in Sri Lanka due to the high costs associated with their development. The use of bio-pesticides in agriculture has, potentially, a similar affect to IPM/IPVM, in that it leads to a reduced reliance on synthetic pesticides and the availability of toxic substances in the community.

**Alter price through taxation or other methods**	Direct influence over price can be achieved through taxation, subsidies or regulation of the price. All pesticides in Sri Lanka currently have a uniform taxation level. Subsidies have been used by the Government of Sri Lanka for other agricultural inputs such as seeds and fertiliser. Direct manipulation of price would try to provide an incentive to farmers to switch to less toxic chemicals and therefore reduce presence and use of more lethal chemicals in the community.

**Safe storage of pesticides at community level**	Safe storage of pesticides has been widely promoted and supported by international organisations and pesticide manufacturers. It has generally entailed the provision of a lockable storage device to limit access at the household level. Several schemes have distributed boxes in Sri Lanka and they have been found to be highly acceptable within communities. No evidence exists yet as to the effectiveness of this strategy in reducing access to pesticides within communities.

**Repackaging of pesticides into non lethal doses**	This option combines several ideas that had previously been suggested together. It involves repackaging specific chemicals into non lethal human doses. The packaging of pesticides is currently mandated under the Control of Pesticides Act and the Registrar of Pesticides is responsible for providing guidance to industry on formulation, size, packaging, name, label, formulation, additives and marketing. These regulations have been used to ensure appropriate safety standards are met. The aim of this option is to limit the concentration or size of packs to non lethal doses which would reduce opportunities for lethal ingestion. Specific safety measures on the packaging could be added to chemicals that were found to be popular choices in self poisoning. An additional benefit of this option is the potential to influence price through the costs incurred to industry for compliance. A recent repackaging of Paraquat following regulations on concentration levels resulted in a doubling of price to the consumer.

**Training pesticide retailers**	Retailers are currently regulated through the Registrar of Pesticides under the Control of Pesticides Act. Dealers must attend a training session and pass a test in order to be a registered dealer. Training currently contains information about safety and storage of pesticides. Additional training modules could be developed to help identify potential high risk customers and develop strategies to reduce access to pesticides for these people.

**Regulation of advertising/marketing**	Regulation of the advertising of pesticides currently sits outside of the Control of Pesticides Act other than the labelling and marketing of products. Limitations have been placed on marketing initiatives and large prizes for substantial sales, are no longer permitted. Further regulation through the use of health promotion strategies for encouraging safe use of pesticides and outlining the harm associated with ingestion could be considered.

### Ratings of Options

**Figure 3:**
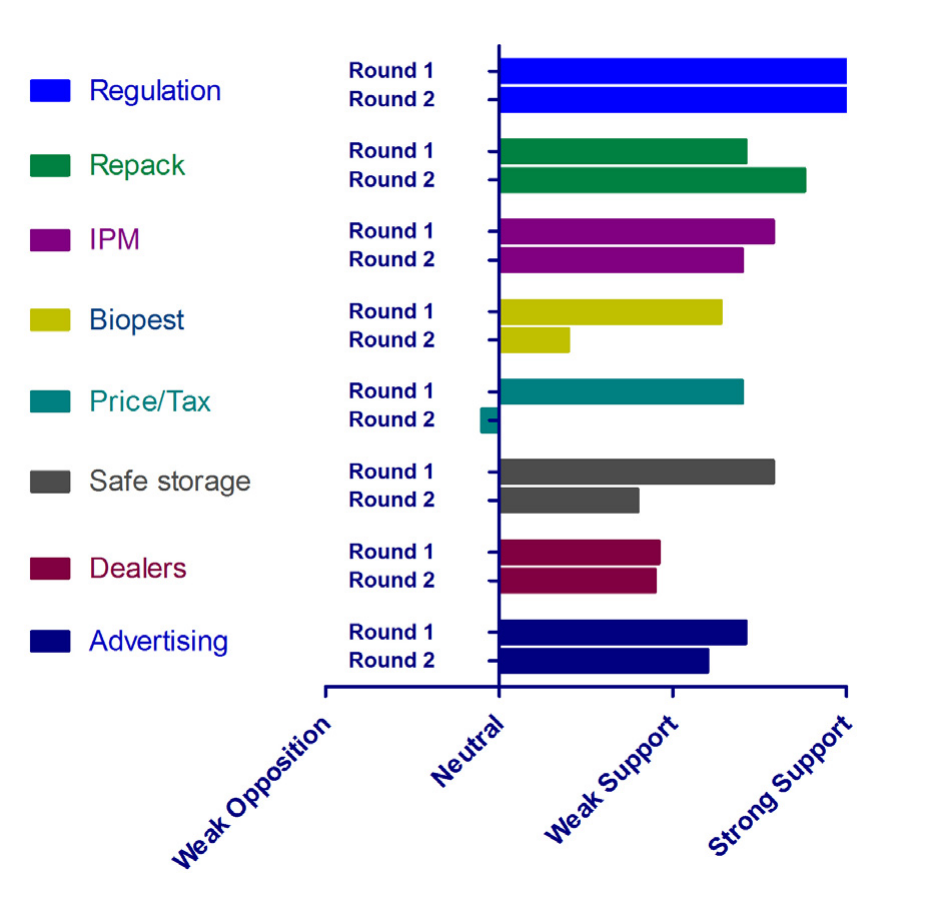
**Comparison of voting between the rounds demonstrating high levels of support for regulation and repackaging**.

The first round of voting generated a high level of support for six of the 8 options: regulation, safe storage, Integrated Pesticide Management (IPM), repackaging, advertising and using bio-pesticides. There were mixed levels of support and opposition to the two remaining policy options, taxation and dealer training programmes. In round one the level of consensus was high in seven of the eight options, with only price and tax incentives being contentious. The discussion between the panellists generated views about the strengths and weaknesses of the various options and has been summarised in Table 3. The second round of voting generated a greater level of differentiation between the options and reflected changes in response to the discussion regarding the desirability of each option. A high level of consensus was only achieved in five of the options. The policy options of regulation and repackaging were more strongly supported than others, with consensus growing for repackaging. This change observed between round 1 and 2 voting can be seen in Figure 3 where support was seen to drop away for IPM, bio-pesticides, price/tax, safe storage, dealer training and advertising. A final ranking of the options seen in Table 3 was initiated by the panellists and identified further regulation and repackaging as the preferred options, followed by IPM, safe storage, advertising, using bio-pesticides, training dealers and taxation.

**Table 3:** Final Ranking of Options

Restriction in access through bans	1
Repackaging of pesticides into non lethal doses	2
Reduce use of pesticides through promotion of IPM	3
Regulation of advertising/marketing	4
Safe storage of pesticides at community level	5
Reduce use through promotion of bio-pesticides	=6
Training pesticide retailers	=6
Alter price through taxation or other methods	8

### Policy Aspects

Through the discussion several themes about policy emerged: options were identified and framed in terms of their strengths and weaknesses, local conditions and contextual factors were highlighted and factors that could modify policy were identified. The panellists went through a subconscious but deliberate process of weighing up the various benefits, harms and costs of each option as seen in Table 4. This process was highlighted by the discussion surrounding price incentives and weighing the impact of political involvement.

**Table 4:** Strengths and Weakness of the identified options

Option	Strengths	Weaknesses
**Regulation**	Strong legislative framework exists. Easy to implement Technical basis strong Past history of success	
		
**Repackage**	Legislation exist Create price variation between more toxic and less toxic products Safe dose for human consumption could be extrapolated Safety measures could be added to product Implementation responsibility of private industry	
		
**Tax**	Price signals can be effective for use in public health	Potential political involvement Politically difficult to implement changes to taxation structures Ministry of Finance and Planning may be against variable taxes on certain products
		
**IPM**	Already current policy supported by Department of Agriculture	Lack of impact on poisoning Doesn't remove pesticides from the home Requires changes to farmer behaviour
		
**Biopesticides**	Could align with other agricultural and environmental priorities	Expensive to implement Needs significant research Lack of impact on poisoning Requires changes to farmer behaviour
		
**Safe storage**	Widely supported by industry and international agencies	Lack of evidence Difficult to implement nationally Changes storage patterns into the home Requires changes to farmer behaviour
		
**Dealer training**	Dealer training programs already in place	Only 20-30% access directly through dealers Lack of impact on poisoning
		
**Advertising**	Current legislation provides some guidance for advertising and marketing	Unclear about what messages could be used Influence of marketing and commission structures impede implementation Dealers very influential in farmer selection

*"You probably could do this but you would probably end up with long protracted battles and perhaps a lot more political involvement than would bring you benefit." *IT 2

Consideration by the panellists of the benefits, harms and costs through the rounds of discussion helped them to make final assessments of their levels of support or opposition to the various options.

Important contextual issues were highlighted by the panellists in relation to the policy process. The delegated authority through the Control of Pesticides Act 1980 to the Pesticide Technical and Advisory Committee (PeTAC) depoliticised many aspects of pesticide control and regulation. This allowed a technical focus for decision making, free of interference from local manufacturing commercial interests.

*"In the Sri Lankan situation...there is no need for us to go further to get policy changes done. We have full freedom to make the decisions. There was never a necessity for us to convince policy makers or politicians." *Ag 1

In addition to the decision making strength of the PeTAC committee the other perceived strengths of the legislation was its ability to have an impact at field level. PeTAC's decisions to withdraw registration for particular products were seen as important previous successes.

*"Evidence for its impact is already in place" *PH1

Strong links between decision making, research and technical advice assisted policymaking. In addition the relationships within agriculture between the public and private sector were strong reflecting close kinship ties generated from links made in the higher education system. These links remained important and the culture of the industry was seen as having been supportive of policy changes. Finally there was a strong belief that policymaking should be informed by evidence, and ongoing surveillance of poisoning was important to continuing the technical basis for decision making.

Several factors were identified as posing threats to some of the potential options. Risks of political interference resulting from pressure from commercial interests and political patronage structures within farming communities were identified. The lack of engagement in pesticide control of the farming communities has strengthened the ability of policymaking but highlighted a potential threat if the issue were to become politicised. Lack of evidence or insufficient evidence of impact was a key factor in the lower ratings accorded to some options, as illustrated by the following quote.

*"We have no evidence of it working yet and until we have evidence of it working; it is hard to push it strongly as a policy" *IT2

The multidimensional context of suicide and the intersectoral action required for solutions highlights the sensitivity of policy to the conflicting interests of each sector. It also made more complex how the problem was defined and what solutions were being proposed. At times this complexity was seen as an issue that those in the health and agriculture sectors could avoid:

*"The social aspects of suicide ... are outside the borders of what pesticides are meant for" *Ag 1.

Difficulties of implementation were often related to weak systems, weak linkages between the sectors and the likely costs. The acceptability of the policy options to the community and the need for behaviour change to accompany policy responses were highlighted as difficulties of implementation at the grass root level. In addition the commission and marketing system for pesticide dealers created market incentives that challenged safe use initiatives and programmes designed to reduce pesticide use.

## Discussion

The themes emerging from the discussion within the epistemic community highlight the complex and multi-dimensional nature of policy. Much policy activity happens under the radar of the grand gestures of high profile speeches and policy documents, and a broader understanding of policy as a "continuing process of social action and interaction" [31] is warranted.

The consideration of policy options within this epistemic community, working on pesticide control to reduce suicide in Sri Lanka, is framed by how they conceived and understood policymaking. The idea of policy within this community is of a rational process of decision making, "*a collective attempt to construct a policy in order to address some evident problem*" [32]. This conceptualisation of policy draws from a 'stages' heuristic that sees policy as a progression through the stages of problem identification, discussion of options, rational choice and implementation. These frames for policy are also apparent in the evidence-based policymaking literature that applies a scientific rational model to decision making. The support and consensus generated for regulation and repackaging of dangerous pesticides was partly based on the delegated authority of the Pesticide Technical Advisory Committee and its ability to access scientific evidence. The support for these options can be understood as reinforcing their ideas and understanding of a linear and rational process through which policy is taken forward.

Despite the intuitive appeal of the rational decision-making frame, the discussion highlighted the limitations of this model in guiding action. Frameworks that incorporate political economy approaches such as the policy triangle and political mapping offer greater explanatory power. "*Policy change is not simply a technocratic process based on rational analysis and that knowledge alone is not sufficient for policy change. Policy is profoundly political*" [33]. A change in the voting patterns between the two rounds of policy option appraisal was heavily influenced by debates on the strength and weaknesses of each option and these were related not just to evidence of effectiveness, but also to considerations of context, the range of actors involved and their likely positions, feasibility of implementation and likely impact.

**Figure 4:**
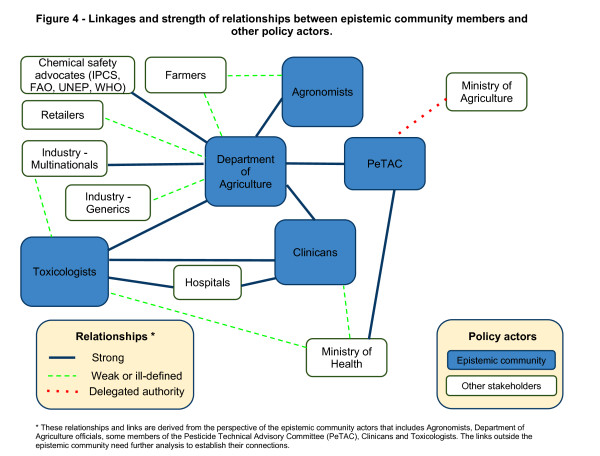
**Linkages and strength of relationships between epistemic community members and other policy actors**.

In the policy analysis triangle, actors are at the heart of the framework and this underlines the importance that individuals, organisations and networks play in influencing policy activity [18]. This exercise has identified a number of the key stakeholders and allows the mapping of the linkages and relationships as described from within this epistemic community. There are strong links and informal connections within this community (Figure 4) that are based on a shared understanding of the problem and long-term engagement in the sector. The relationships between members of the epistemic community and outside actors are mostly divided along sectoral lines (health and agriculture). The relationships between the actors outside this community need further exploration to understand their influence on policymaking and to frame the options beyond those generated by this epistemic community.

The 'problem' was perceived in this community in three main ways: people swallowing pesticides (clearly not their intended use), lack of evidence for interventions that have an impact on mortality, and problems of implementation. These narratives of the problem point to a more complex interplay of factors that determine the relative suitability of options rather than simply their content. Within this local context the consensus and support for the options of regulation and repackaging were perceived to be more suitable because they reduced access to means, there was strong evidence that they could work and the legislation and authority existed for their implementation.

Knowledge and evidence are the key features of epistemic communities and therefore closer examination of the flow of knowledge, status of the evidence and its role within this community is useful. The majority of the evidence came from two sources, plant protection research and poisoning research. These two areas of research have direct linkages into the Pesticide Technical Advisory Committee and in turn this strengthened the ties between the epistemic community members and 'institutionalises their influence' [25]. The status of the evidence base was strong and much was known about the choice of poison from anecdotal sources as well as evidence from surveillance. This use of epidemiological data and clinical experience created strong links between the health actors and provided a solid foundation for advocacy within, and from, the epistemic community to the broader social policy domain.

Interaction between the epistemic community and policy makers is needed "to ensure knowledge generation is policy relevant"[34]. The linkages between the knowledge generators and the policymakers were strong and integral to this community and served a mutually reinforcing function. Knowledge and evidence was generated and interpreted within the community and placed on the agenda with decision makers. These linkages served to reinforce the boundary within the community and ensured that evidence informed policymaking was maintained [35]. However while this epistemic community is influential and well linked the solutions they generate are limited to ones that supported, or at least fitted comfortably within the status quo. The solutions they proposed offer the opportunity to change things from within agreed values, discourse and interests [36]. If more radical change is needed or desired then this community would need to engage in a wider process to generate support for such options.

This exercise exposed potential policy options that would garner support from this influential and powerful epistemic community and is akin to the deliberative process advocated for by Culyer and Lomas [37]. It produced outcomes that have been suggested as important features of deliberative processes including the contextualisation of proposed interventions, identified relevant clinical, social and political influences, conferred credibility, and identified impediments to implementation.

In addition to knowledge and evidence generated from within this community there is a need to consider other areas of influence on policymaking, as highlighted by Bowen and Zwi [21]. The SUPPORT tools also help to provide some guidance on additional areas that are necessary when framing options: technical feasibility fits with dominant values, acceptable in terms of budget and acceptability to stakeholders [38]. Further exploration is needed of the acceptability of the options to stakeholders, likely costs and cost-effectiveness in order to generate consensus beyond this community.

### Limitations of the study

A limitation of this study was the number and range of panellists available to participate. However, a number of studies have shown that panel size is not always a good indicator of the success of a consensus exercise [29,30]. The consideration of panel size can only be understood in relation to the ability of the exercise to produce innovative ideas. In this case study, the process produced a new policy option that blended together, in an innovative way, several previously considered options. The advantages of having the face to face group process were that it enabled a robust discussion of the options and a thorough examination of the strengths and weaknesses of each. The limited range of experts meant that the panellists were familiar with the background literature and the main proposed solutions and so the discussion was able to be more intensive and focussed, with limited defensiveness on the part of the participants given the high levels of continuity and trust that operated within the community.

## Conclusions

The conceptualisation of policy as multi-dimensional, which includes aspects of context, networks, knowledge, implementation and impact, influenced how the participants considered and rated the options. Development and consideration of policy options to reduce access to pesticides in Sri Lanka generated the strongest support and consensus for two options: further regulation of pesticides and repackaging of the most toxic formulations into safe doses. The consensus and support for these policy options reflects an understanding by the panellists of the factors that facilitate or thwart policy change within this specific problem and context. Further examination of the potential barriers and opportunities for the options identified will help ascertain the best strategy for implementation.

Engaging with the epistemic community provided a valuable insight into many important aspects of policymaking. The exercise provided an opportunity to identify the status of the evidence, debate the strengths and weaknesses of future policy options, and make judgements about trade-offs. The observed discussion revealed insights into how this community operates - the linkages and relationships within and outside of the community and the status and role of evidence and knowledge. Use of epistemic community actors revealed layers of contextual analysis that inform policymaking, even in ostensibly technical areas.

## Competing interests

NB and MP work with the South Asian Clinical Toxicology Research Collaboration that aims to reduce deaths from pesticide poisoning through regional collaboration.

## Authors' contributions

MP undertook the fieldwork, analysis and wrote the first draft of this paper. AZ provided guidance throughout in study design, conduct, analysis and writing of the paper. NB holds the grant under which this research has been funded, facilitated the exercise described, and contributed to write-up and analysis. All authors contributed to writing and agreed the final text.

## Authors' information

MP is a research degree student supervised by AZ and co-supervised by NB and Dr Duncan McDuie-Ra.
